# Effects of generative artificial intelligence on students' learning motivation: evidence from a meta-analysis

**DOI:** 10.3389/fpsyg.2026.1832451

**Published:** 2026-09-24

**Authors:** Longyue Fang, Zhenzhi Li, Bin Xiong, Zhuotao Lu

**Affiliations:** 1 School of Mathematical Sciences, East China Normal University, Shanghai, China; 2 Shanghai Key Laboratory of Pure Mathematics and Mathematical Practice, East China Normal University, Shanghai, China; 3 School of Education Science, Qingdao University, Qingdao, Shandong, China

**Keywords:** generative artificial intelligence, meta-analysis, moderator analysis, motivation, self-determination theory

## Abstract

**Background:**

The use of generative artificial intelligence (GenAI) in the education setting has expanded quickly. GenAI may enhance learning motivation by providing immediate feedback, adaptive support, and multimodal resources, yet empirical findings remain inconsistent.

**Aims:**

To address the controversy of current studies, a meta-analysis was conducted to examine the overall effect of GenAI on students' learning motivation and test whether effects differed across study contexts.

**Methods:**

A random-effects meta-analysis synthesized 56 effect sizes from 42 controlled experimental and quasi-experimental studies involving 6,059 participants. Moderator analyses assessed variation by educational level, cultural context, subject domain, and device type.

**Results:**

The random-effects model showed a positive average effect of GenAI on students' learning motivation (Hedges' *g* = 0.764). However, heterogeneity was very high (*I*^2^ = 93.7%), indicating substantial variation across study contexts. Exploratory moderator analyses suggested that effects were larger in higher education than in primary/secondary education, larger in collectivistic than individualistic contexts, larger in language learning than in other subjects, and larger when GenAI was used on mobile devices (MD) rather than computers. The observed subgroup differences should be interpreted cautiously because these moderators represented study-level characteristics rather than experimentally manipulated conditions.

**Conclusions:**

GenAI-supported learning is associated with higher learning motivation on average; however, the substantial heterogeneity suggests that its motivational benefits are not uniform across educational contexts. Future research should identify the instructional, learner, and contextual conditions under which GenAI can most effectively support students' motivation.

## 1 Introduction

Generative artificial intelligence (GenAI) refers to AI systems that can create new content including text, images, or code, rather than solely processing or classifying existing information (Sengar et al., 2025). The public release of ChatGPT in late 2022 constituted a watershed moment in the mainstream visibility of such a system. In the year since, large language models and allied GenAI systems have undergone rapid capability expansion, demonstrating increasingly sophisticated competence in natural language interpretation, multimodal output generation, and real-time interactive feedback (Alier et al., 2024).

These affordances carry significant pedagogical implications, enabling learning experiences that are more adaptive, individualized, and responsive to diverse learner profiles (Bolanos et al., 2024). Indeed, the penetration of GenAI into formal education has been correspondingly broad. GenAI can leverage AR and VR technologies to enrich immersive learning (Liu X. et al., 2025), tailor personalized explanations and practice activities aligned with students' needs and cognitive levels (Chiu et al., 2024), and use video-generation tools to translate abstract concepts into vivid visual representations (Abdelfattah et al., 2025). Across subject domains, applications include synthesis of clinical cases to scaffold medical knowledge study (Afzaal et al., 2025), serve as a conversational partner or writing coach for language learning (Pack and Maloney, 2023), and generate code with accompanying annotations to facilitate learning in information technology (Cubillos et al., 2025). Beyond specific subject content, GenAI-based intelligent learning companions (Cain, 2025), personalized intelligent teachers (Reddig et al., 2025), and intelligent voice-interaction systems (Young, 2024) are reshaping traditional classroom ecologies and redefining teacher–student role boundaries, and signaling a future shift from teacher-centered instruction toward human–AI collaborative learning.

Learning motivation refers to the internal psychological process that initiates and sustains learning behaviors, reflected in learners' goal orientations, value appraisals, and willingness to invest effort in academic tasks (Pintrich, 2003). Accumulating evidence identifies motivation as a central driver of academic success (Diseth, 2025). Higher motivation is associated with better achievement (Feng et al., 2013; Steinmayr et al., 2019), and students who begin a new academic year with stronger motivation tend to demonstrate greater subsequent progress (Dai et al., 2026), resulting from the tendency to adopt deep learning strategies that strengthen retention and promote meaningful engagement among those students (Kamberi, 2025). Overall, given the correlation between motivation and higher levels of accomplishment (Huang et al., 2025), a systematic investigation of GenAI's effects on motivational processes is both theoretically significant and practically consequential for efforts to harness these technologies in the service of more effective and equitable learning.

## 2 Problem statement

Given the central role of motivation in shaping students' learning engagement and academic achievement, understanding how GenAI influences learning motivation has become an important educational and research concern. GenAI offers several features that are theoretically conducive to motivation, including personalized support, immediate feedback, adaptive scaffolding, and interactive learning experiences (Alenezi and Alenezi, 2025; Hmoud et al., 2024; Shao, 2025). At the same time, the findings remain fragmented, with studies reporting positive, weak, null, and occasionally negative effects (Podoliak et al, 2025; Zhang, 2025; Heung and Chiu, 2025). Moreover, substantial variation exists across studies in terms of educational level, subject domain, intervention characteristics, technological functions, and cultural settings. Such variation suggests that the motivational effects of GenAI may depend on specific contextual and design conditions. A systematic quantitative synthesis is therefore needed to establish the overall relationship between GenAI and learning motivation and to identify the factors that may explain differences in observed effects across studies, so as to give robust evidence for educators and policymakers to decide whether, when, and how GenAI should be adopted to support students' motivation.

## 3 Generative artificial intelligence and student motivation: overview

### 3.1 Theoretical associations between GenAI and learning motivation

Empirical studies across different fields have reported similar patterns suggesting that GenAI can improve students' motivation. In programming, environment supported by large language model It actually goes beyond specific subject content may satisfy students' basic psychological needs, leading to higher autonomous motivation and improved programming competence (Choi and Kim, 2025). In foreign language learning, Iranian EFL students receiving ChatGPT-assisted instruction outperformed controls in writing accuracy and learning motivation (Jafari, 2025), and similar benefits have been reported when ChatGPT is integrated into writing instruction (Song and Song, 2023). Beyond writing, process-oriented AI tools have also shown motivational advantages. An 8-week intervention with Chinese university language learners indicated that Duolingo and related AI tools increased motivation by enhancing perceived control, reducing anxiety, and strengthening self-efficacy (Shao, 2025). Consistently, 10- and 12-week Duolingo-based GenAI interventions likewise reported increased motivation for foreign language learning (Wei et al., 2023; Yuan and Liu, 2025).

Evidence suggests that GenAI may enhance students' learning motivation and outcomes by creating a more accessible, responsive and personalized learning environment. This pattern is consistent with self-determination theory (SDT), which distinguishes intrinsic motivation (IM), rooted in interest and curiosity, from extrinsic motivation (EM), driven by external contingencies such as rewards and grades (Ryan and Deci, 2000). Through internet access, students can consult GenAI anytime and anywhere to obtain immediate answers or tutoring (Rahman and Watanobe, 2023). Its continuous availability lowers barriers to help-seeking and enables learners to progress at their own pace, fostering interest, enjoyment, and satisfaction, which are the key indicators of intrinsic motivation (Legault, 2020). GenAI can tailor responses to learners' inputs, enabling scalable personalized instruction that has historically been difficult to implement (Mohamed et al., 2025). This individualized scaffolding accommodates diverse learning preferences, increases engagement and interest, and supports deeper understanding and confidence, thereby strengthening intrinsic motivation (Alenezi and Alenezi, 2025; Qu et al., 2024; Shute, 2008). GenAI's interactivity may further amplify these benefits. Unlike static textbooks, AI chatbots allow learners to ask questions and receive iterative explanations; when concepts are unclear, students can request analogies or step-by-step decompositions and obtain immediate formative guidance (Ng et al., 2024). Such dialogic interaction can enhance perceived competence and autonomy by enabling inquiry and instant clarification (Annamalai et al., 2023). Through dynamic interaction and adaptive support, GenAI may make learning more engaging and less burdensome, further increasing intrinsic motivation (Hew et al., 2023).

Although GenAI may also reinforce extrinsic motivation by highlighting external rewards, such as future income, praise, or career opportunities (Hmoud et al., 2024), existing evidence suggests that benefits of GenAI appear to be more strongly related to intrinsic rather than extrinsic motivation. For example, (Huang and Mizumoto 2024) found that integrating ChatGPT into an English writing course significantly enhanced learners' intrinsic motivation (e.g., pleasure and engagement in writing) but did not significantly change obligation-driven motivation. In addition, (Wu et al. 2024) assessed the effects of a ChatGPT-based intelligent learning support tool on two types of motivation among high school students and found that gains in intrinsic motivation far exceeded those in extrinsic motivation. Despite its potential benefits, GenAI may also impose motivational costs through prolonged screen exposure, redundant output, hallucinations, and excessive convenience. Extended screen viewing can increase students' visual fatigue (Kaur et al., 2022) leading to higher stress and anxiety (Issa et al., 2023). Moreover, GenAI responses are often more verbose than human answers and may overwhelm learners with redundant or irrelevant information, diverting attention from core concepts (Kabir et al., 2024). Such information overload can crowd learners' limited working memory with extraneous details, producing excessive cognitive load (Torcasio and Sweller, 2010) and dampening enthusiasm for learning.

GenAI hallucinations pose an additional risk by exposing students to misinformation and fostering inaccurate understandings, which have been shown to reduce users' overall attitudes toward GenAI (Lee et al., 2025). When no valid answer exists, GenAI may fabricate plausible-sounding responses to satisfy the prompt (Wach et al., 2023). Empirical evidence suggests that only about 20% of students can identify AI-generated factual errors, implying that many may unknowingly incorporate false information into their knowledge structures (Dang and Nguyen, 2025). To manage these errors, learners may need to expend additional effort cross-checking sources, increasing the time costs of learning and potentially creating an unfavorable learning experience that undermines motivation.

Finally, GenAI's convenience may encourage overreliance and reduce learner autonomy, thereby weakening motivation (Heung and Chiu, 2025), cognitive engagement and critical thinking (Alhur et al., 2025). Additionally, prior users of GPT can perform worse than peers who never used it when AI support is removed (Bastani et al., 2025). Over time, such dependency may diminish problem-solving competence and reduce students' willingness to invest in learning tasks.

### 3.2 Potential moderators

#### 3.2.1 Study characteristics

GenAI's effects on learning motivation may vary as a function of intervention duration. On one hand, longer interventions allow students to gradually adapt to a new technology and accumulate more substantial positive effects (Kim and Kim, 2025). For example, a 7-month longitudinal study reported that sustained use of GenAI-assisted instruction partially satisfied students' psychological needs and helped maintain their learning enthusiasm (Galindo-Domínguez et al., 2025). On the other hand, once users become accustomed to a product, the novelty effect of a new technology may diminish (Jeno et al., 2019), and motivational gains may decline accordingly. A 10-week study found that students who learned with ChatGPT reported lower motivation for learning English than their peers in the control group (Yıldız et al., 2023). Therefore, whether intervention duration moderates the impact of GenAI on learning motivation remains unclear.

GenAI effects may also differ across publication years. The sophistication of GenAI has continued to advance over time. Taking OpenAI as an example, from the launch of ChatGPT-3 to the emergence of ChatGPT-5, its capabilities in logical reasoning, programming tasks, and semantic understanding and the quality of generated responses have increased substantially within 3 years (Sanli et al., 2025). Such functional improvements may provide students with better learning experiences (Ma and Zhong, 2025), thereby more strongly promoting motivation. Subject domain may also moderate the effect of GenAI on learning motivation. Disciplinary knowledge and the required competencies vary a lot among subjects. For instance, mathematics emphasizes logical thinking, formal proof, and computation (Biehler et al., 1994), whereas language learning relies on semantic comprehension, memory, and necessary pronunciation practice (Johnson, 2017). In science-related subjects, experimentation plays an indispensable role, requiring students to develop hands-on practical skills and an inquiry orientation, as well as the capacity to use learned theories to guide practice (Shneider, 2009). Given these disciplinary distinctions, the benefits of using GenAI may not be uniform across subjects; therefore, the present study includes subject domain as a moderator.

#### 3.2.2 Demographics

Education stage may moderate GenAI's effects on learning motivation, as cognitive development and responses to emerging technologies vary by age. Exploratory tendencies tend to decline with age (Nussenbaum et al., 2023), suggesting that younger learners may more readily experience curiosity and interest when encountering novel tools. In the field of language, studies in primary school report that GenAI can enhance writing motivation (Liu et al., 2024), while a study in secondary school found that GenAI-supported game-based learning increased intrinsic motivation, but the improvement was modest (Chen and Chang, 2024). In contrast, a meta-analysis found no significant differences in GenAI effects across educational stages (Liu X. et al., 2025). Overall, the evidence remains mixed, warranting further tests of education stage as a moderator.

Gender may also moderate the impact of GenAI on learning motivation. Prior research suggests that males tend to hold more favorable attitudes toward new technologies (Cai et al., 2017) and may therefore engage more proactively with AI tools. Similarly, male students across multiple universities in Sweden held more positive attitudes toward using ChatGPT, whereas female students expressed more concerns (Stöhr et al., 2024). In the context of learning AI-related courses, boys in Beijing primary schools demonstrated stronger interest, confidence, and willingness to participate than girls (Lin et al., 2021). Moreover, male researchers reported prolonged and more frequent GenAI use and greater efficiency gains than their female counterparts (Tang et al., 2025). Taken together, GenAI use and its potential benefits may vary by gender. Students' cultural background may moderate GenAI's impact on learning motivation. Cultural psychology argues that cultural contexts shape psychological processes and learning behavior (Ratner, 2002). Following Hofstede's cultural dimensions, we classify contexts as collectivist vs. individualist (Khlif, 2016). In collectivist cultures where group goals and mutual support are more valued (Harvey, 1997), GenAI may be more readily adopted when positioned as a collaborative tool for learning with teachers and peers. Consistent with this view, studies from collectivist countries such as China and India suggest that GenAI use can significantly enhance learning motivation (Dou and Sun, 2025; Suciati et al., 2024). By contrast, in individualist cultures, learners may emphasize and respond more strongly to the personalized and autonomy-supportive features of GenAI, thus fostering intrinsic or autonomous motivation. Accordingly, a GenAI-based programming environment in Korea increased first-year high school students' autonomous motivation and programming competence (Choi and Kim, 2025). Therefore, we examine cultural background as a moderator.

#### 3.2.3 GenAI characteristics

GenAI's effects on learning motivation may vary by device type. In this study, GenAI device type was coded as mobile devices (MD) vs. PCs. The portability of mobile devices enables learning to occur anytime and anywhere, which may increase learning engagement and motivation by supporting immediate interaction and greater accessibility (Garzón et al., 2025). However, mobile devices may also introduce additional distractions. When students use laptops for educational purposes, learning is often associated with fewer interruptions and stronger academic focus. By contrast, learning on smartphones is more frequently interrupted by social media notifications and other disruptions, which can undermine concentration and sustained motivation (Abdelghany, 2025). Accordingly, we include GenAI device type as a moderator.

GenAI function may also moderate the link between GenAI use and learning motivation. Following a prior meta-analysis, GenAI function can be classified as assessment (evaluating student performance), personalized learning support (PLS) and intelligent tutoring systems (ITS) (Zhu et al., 2025). Among these, personalized support may be particularly beneficial because it can adapt content and pacing to students' needs, satisfy psychological needs, and thereby enhance motivation (Wu et al., 2024). By contrast, evidence on intelligent tutoring systems remains less consistent, with some studies finding limited gains in broader learning outcomes (Liao et al., 2024). The present study adopts the same scheme to test whether GenAI function moderates GenAI's effects on learning motivation.

### 3.3 Research gap, research questions and hypothesis

Recent educational technology research has generated substantial meta-analytic evidence indicating broadly positive effects of generative artificial intelligence (GenAI) in educational settings. For example, ChatGPT has been associated with better academic outcomes, affective–motivational states, and dispositions toward higher-order thinking (Deng et al., 2025; Wang and Fan, 2025; Zhao et al., 2025; Nie et al., 2025; Mo et al., 2026). Beyond these outcomes, syntheses also document gains in academic achievement (Han et al., 2025; Sun and Zhou, 2024; Liu Z. et al., 2025), creativity (Holzner et al., 2025), computational thinking (Cheng and Hu, 2025), and STEM learning outcomes (Li et al., 2025). However, most existing syntheses have focused on performance- and skill-related outcomes, whereas learning motivation, as a fundamental academic-affective construct, remains insufficiently synthesized. To date, no meta-analysis has specifically examined GenAI's effects on learning motivation.

To address this gap, the present study focuses on motivation. Specifically, the present study conducts a meta-analysis to estimate the overall effect of GenAI on students' learning motivation and to examine whether this effect varies by GenAI characteristics, study characteristics, and demographic factors. In doing so, the study aims to clarify not only whether GenAI is motivationally beneficial, but also under what conditions its effects are stronger or weaker. Accordingly, this study seeks to answer two research questions:

a What is the effect of GenAI on students' learning motivation?
b Which factors moderate GenAI's effect on students' learning motivation?

Based on the previous literature review, we put forward the following hypotheses:

H-1a. Students using GenAI have more intrinsic motivation.

H-1b. Students using GenAI have more extrinsic motivation.

H-1c. GenAI shows a larger effect on intrinsic motivation than extrinsic motivation.

H-2. The link between GenAI and learning motivation is stronger in later years.

H-3. The link between GenAI and learning motivation is stronger for male students than for female students.

## 4 Research method

Meta-analysis aggregates statistically comparable effect sizes from independent empirical studies to derive a summary effect, assess the magnitude of between-study heterogeneity, and examine potential moderators that account for variation in findings (Cooper et al., 2019). Methodologically, a meta-analysis is appropriate for the present study because the research problem concerns a growing but inconsistent body of quantitative intervention evidence rather than a single empirical case or a purely conceptual issue. Individual studies have examined the effects of GenAI on students' learning motivation across different learner groups, subject domains, cultural contexts, devices, and implementation conditions, but their findings vary in direction and magnitude. A narrative review alone would be insufficient to determine the magnitude and precision of the overall effect or to assess whether such variation reflects systematic contextual differences. Because the eligible studies used experimental or quasi-experimental comparisons and reported quantitative motivation outcomes, their findings can be converted into a common standardized effect-size metric, allowing cross-study synthesis. This methodological approach therefore aligns with the aims of the present study: to estimate the overall effect of GenAI on learning motivation, quantify between-study heterogeneity, and examine whether study characteristics, learner demographics, and GenAI-related features explain variation across effects.

### 4.1 Literature search

The release of GPT-based large language models on November 30, 2022, marked a major public-facing moment in the widespread adoption of generative artificial intelligence. Given the lag between study completion and publication, it is unlikely that relevant empirical studies could have been published within 2022. Accordingly, studies published between January 2023 and November 2025 were searched. The search strategy was designed to identify controlled studies examining GenAI-supported learning and quantitatively assessed learning motivation. The search procedures followed the PRISMA 2020 reporting guideline (Page et al., 2021) and were conducted in major academic databases and platforms, including Web of Science, ERIC, ProQuest, EBSCO, and Scopus. Our search string was designed to capture studies investigating the impacts of GenAI on students' motivation and consisted of the following: (“generative artificial intelligence” OR “generative AI” OR “GenAI” OR “ChatGPT” OR “GPT” OR “large language model” OR “LLM” OR “chatbot” OR “AI chatbot” OR “AI tutor” OR “AI tutoring” OR “artificial intelligence” OR “AI”) AND (“learning motivation” OR “motivation”). The initial database search yielded 2,365 records. A second-round search using citation chasing (i.e., reviewing the reference lists and citing articles of the retrieved studies) identified an additional 74 records. After removing 541 duplicates, 1,898 records remained. Eligible sources were limited to studies that were published or accepted for publication that were publicly accessible and provided sufficient quantitative data for effect-size calculation. These sources included peer-reviewed journal articles and published full conference papers. The complete database-specific search strategies are provided in **Table 5** in **Appendix A**.

### 4.2 Inclusion criteria

Studies were included if they met the following criteria:

1. Population: Participants were students enrolled in formal educational settings, ranging from primary school to university.
2. Intervention: The experimental condition involved learning or instruction supported by generative artificial intelligence, such as large language model-based chatbots, GenAI tutors, or systems that generated instructional content, explanations, feedback, or adaptive learning support. GenAI had to constitute an identifiable component of the intervention.
3. Comparator: The comparison group studied the same or closely related content without GenAI support, including conventional instruction or non-GenAI-supported digital learning.
4. Outcome: Learning motivation was assessed quantitatively using a scale, subscale, or composite score explicitly described by the original authors as learning motivation, academic motivation, motivation to learn, intrinsic motivation, extrinsic motivation, or a closely related motivational construct.
5. Study design: Studies employed a controlled experimental or quasi-experimental design that included both a GenAI-supported group and a non-GenAI comparison group.
6. The study reported sufficient statistics to compute effect sizes, including sample size, mean(s), and standard deviation(s).
7. The article was written in English.

The PICOS framework is shown in Table 1. Applying these criteria, 42 eligible studies (56 effect sizes) were ultimately included in the meta-analysis. The study selection process comprised four stages—identification, screening, eligibility, and inclusion—as shown in Figure 1.

**Table 1:** PICOS framework and eligibility criteria.

PICOS	Include	Exclude
Population	Students enrolled in formal educational settings from primary school through university	Teachers, non-student populations, informal learners, or participants whose educational status could not be determined
Intervention	Learning or instruction supported by generative artificial intelligence, including large language models, GenAI chatbots, AI tutors, or other GenAI systems capable of generating content, feedback, explanations, or adaptive learning support	Conventional artificial intelligence without generative functions, administrative uses of GenAI, or multicomponent interventions in which the effect of GenAI could not be isolated
Comparator	A comparable learning condition in which students studied the same or closely related content without GenAI support, including conventional instruction, non-GenAI digital learning, or other eligible control conditions	Studies without a comparison group or studies in which both groups received equivalent GenAI support
Outcome	Quantitatively assessed learning motivation, including learning motivation, academic motivation, motivation to learn, intrinsic motivation, extrinsic motivation, or a general/combined learning-motivation score explicitly identified by the original authors	Engagement, interest, satisfaction, attitude or anxiety without a separately reported eligible motivation score
Study design	Controlled experimental or quasi-experimental studies that included both a GenAI-supported group and a non-GenAI comparison group	Single-group, correlational, qualitative, descriptive, review, or conceptual studies

**Figure 1:**
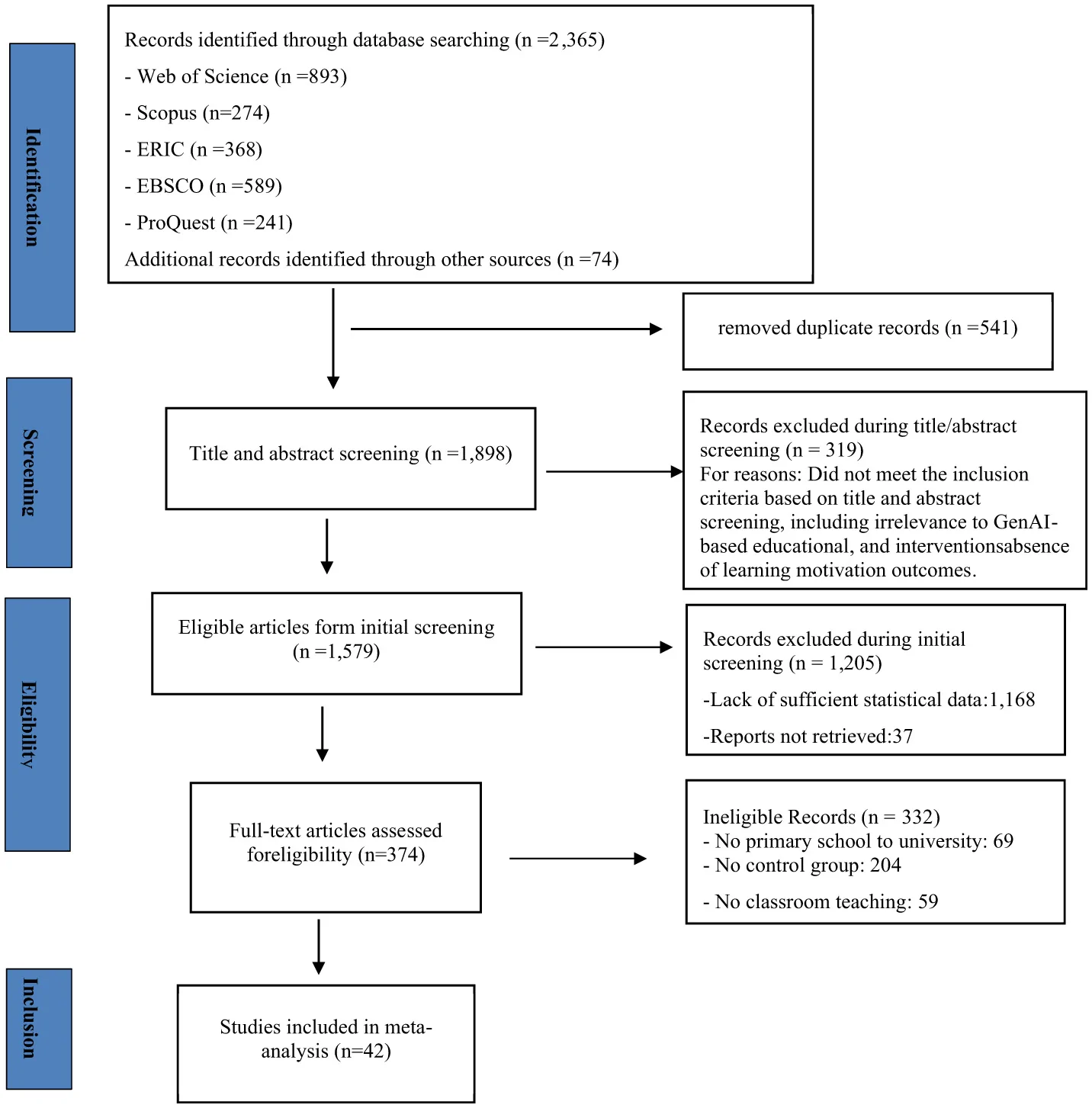
Flow diagram of literature search and study inclusion criteria.

### 4.3 Study quality evaluation

To characterize the methodological quality of the included studies and inform the interpretation of the meta-analytic findings, the Basic Quality Assessment of Primary Study (BQAPS; Xu et al., 2024) was adopted. The BQAPS is based on the Cochrane risk-of-bias framework and contains 12 items covering study design, measurement reliability and validity, statistical procedures, and key aspects of intervention implementation. Each item is scored on a three-point scale: 2 points if the criterion is fully met, 1 point if it is partially met, and 0 points if it is not met. Total scores range from 0 to 24, with higher scores indicating stronger methodological quality and a lower risk of bias. The first and second authors independently rated all included studies. Agreement for the item-level ratings was high (Cohen's κ = 0.83). Disagreements were resolved through discussion and re-examination of the original reports. Total BQAPS scores ranged from 18 to 23 (*M* = 20.38), indicating that the included studies ranged from medium-high to very high methodological quality according to the interpretive categories proposed by Xu et al. (2024). No study was excluded solely on the basis of its BQAPS score. The BQAPS scores for each included study are presented in **Table 6 of Appendix A**.

### 4.4 Literature coding

All articles included in the meta-analysis were coded for their key study components. The first and second authors independently coded all studies and extracted posttest means, standard deviations, and sample sizes for the experimental and control groups to compute effect sizes (Hedges' *g*). Each independent sample was assigned a separate effect size. When a study reported multiple independent samples, each sample was coded separately. When studies involved multiple experimental groups or reported outcomes under different moderator conditions, data were extracted for each distinct comparison condition. If a study reported only subdimension-level motivation outcomes without an overall motivation score, subdimension data were imported into CMA 3.0 and aggregated to generate an overall Hedges' *g*-value representing total motivation. After coding was completed, inter-coder agreement was evaluated using Cohen's kappa. The resulting Cohen's kappa was 0.91, indicating acceptable reliability (Viera and Garrett, 2005). Any discrepancies were discussed, with each coder providing justification, until consensus was reached.

The following study characteristics were coded (coding schemes are presented in parentheses):

Study ID: author(s) and publication year (when a single study reported multiple results, they were distinguished in numerical order; for studies with more than three authors, letters were appended after the year to differentiate publications with the same first author and year).

Cultural background: collectivist (Collect), individualist (Individ).

Education stage: primary school (Primary), secondary school (Second.), university (Univ.).

GenAI function: assessment (AST), intelligent tutoring systems (ITS), personalized learning support (PLS).

GenAI device type: personal computer (PC), mobile device (MD).

Intervention duration: ≤ 4 weeks (short), >4 and ≤ 8 weeks (medium), >8 weeks (long).

Gender: proportion of females in the experimental group.

Motivation type: intrinsic motivation (IM), extrinsic motivation (EM).

The final coded dataset comprised 56 effect sizes, as shown in **Table 7 in Appendix A**. In the coding table, “NA” indicates missing information.

### 4.5 Coding of motivation outcomes

Motivation coding was guided by self-determination theory, which distinguishes intrinsic motivation from extrinsic motivation (Ryan and Deci, 2000). In this meta-analysis, learning motivation was defined as students' motivational orientation toward learning tasks, reflected in their interest, enjoyment, curiosity, willingness to invest effort, perceived value of learning, goal-directed striving, or externally regulated reasons for learning such as grades, rewards, obligations, social recognition, or career-related benefits.

Motivation type was coded only as intrinsic motivation or extrinsic motivation. Intrinsic motivation refers to outcomes reflecting interest, enjoyment, curiosity, autonomous engagement, or satisfaction derived from the learning activity itself. Extrinsic motivation refers to outcomes reflecting motivation driven by external incentives or pressures, such as grades, rewards, praise, social expectations, obligation, or future academic and career benefits.

Some primary studies reported general, positive, total, or combined motivation scores rather than separate intrinsic and extrinsic motivation scores. These outcomes were included in the overall analysis when they measured students' learning motivation, but they were not treated as a separate motivation type. Instead, they were coded as general or overall learning motivation and excluded from the intrinsic-vs.-extrinsic subgroup analysis unless the scale content or subscale labels allowed a clear classification as intrinsic or extrinsic motivation. When a study reported both a total motivation score and separate intrinsic/extrinsic subscale scores, the total score was used for the overall motivation estimate, whereas the subscale-specific scores were used for the motivation-type subgroup analysis.

### 4.6 Meta-analysis process

#### 4.6.1 Effect size and model selection

Post-test between-group SMD is a widely adopted effect-size index for comparative intervention meta-analyses because it directly estimates the standardized difference between experimental and control groups after intervention (Hedges and Olkin, 2014; Lipsey and Wilson, 2001). This approach has been commonly used in educational and psychological meta-analyses (Cooper et al., 2019; Borenstein et al., 2021), including recent meta-analyses relying on post-test data alone (Dahl-Leonard et al., 2024; Wijnia et al., 2024; Juhász et al., 2024). Moreover, pre-test–post-test change effects require reliable pre-test–post-test correlations, which were inconsistently reported in the included studies; estimating these correlations may introduce additional uncertainty into effect-size calculations (Borenstein et al., 2021). Therefore, post-test scores were used as the common metric to ensure comparability across studies. Hedges' *g* was adopted as the standardized mean difference statistic, as it provides a small-sample bias–corrected version of Cohen's *d* and is widely recommended as an unbiased estimator for continuous outcomes in meta-analyses (Goulet-Pelletier and Cousineau, 2018; Lin and Aloe, 2021).

A fixed-effects model assumes that all included studies share a single true effect size and that any observed between-study differences are entirely attributable to sampling error. In contrast, a random-effects model assumes that true effect sizes vary across studies and that these effects follow a normal distribution (Dettori et al., 2022). In the present study, between-study heterogeneity was assessed using Cochran's *Q*-test and the *I*^2^ statistic. Cochran's *Q* evaluates whether heterogeneity is statistically significant, whereas *I*^2^ quantifies the proportion of total variability attributable to true heterogeneity. When heterogeneity was substantial (*I*^2^ > 50%), a random-effects model was adopted; otherwise, a fixed-effects model was used (Deeks et al., 2019).

Given that multiple effect sizes were derived from individual studies, an additional three-level meta-analysis was performed to examine whether potential dependence among effect sizes materially affected the pooled estimates. All data analyses for the three-level meta-analysis were conducted using R software (version 4.3.2). The analyses were implemented using the metafor package, following the R programming manual by Assink and Wibbelink (2016). In this model, sampling variance was specified at Level 1, effect sizes at Level 2, and studies at Level 3. This structure allowed effect sizes to be nested within studies and provided a way to evaluate whether substantial variance existed within studies in addition to variance between studies. Three-level meta-analysis has been recommended as a way to model dependent effect sizes when multiple effects are extracted from the same primary studies (Nie et al., 2025). The full three-level model was compared with reduced models in which the Level 2 or Level 3 variance component was constrained to zero, using likelihood-ratio tests. This analysis served as a sensitivity check to determine whether retention of the full three-level structure was warranted. Aggregation of all effect sizes into a single estimate per study was not adopted as the primary analytical strategy, as averaging multiple effects within a study may remove valuable within-study variation and obscure moderator-relevant differences across outcome types, intervention conditions, or coding categories (Cheung, 2019), especially when multiple effects represent substantively distinct motivation outcomes, intervention conditions, or coding categories. However, the possibility of residual dependence is addressed in the Limitations section.

#### 4.6.2 Data processing and analysis

The meta-analysis was conducted using CMA 3.0. For continuous variables (gender), meta-regression analysis was employed. For categorical variables, subgroup analysis was performed to identify and interpret the sources of heterogeneity. In line with common methodological practice, pooled effects were only computed for subgroups comprising at least two independent studies (Rodríguez-Sánchez-Laulhé et al., 2024). Studies without available data for the moderator variables were excluded from the corresponding subgroup analyses.

## 5 Results

This meta-analysis examined 56 effect sizes in 42 studies of 6,059 participants from 2023 to 2025. Sample sizes ranged from 32 to 486.

### 5.1 Statistical model fit and comparative analysis

To address the possibility that multiple effect sizes from the same study were statistically dependent, we conducted a supplementary three-level meta-analysis. The variance decomposition showed that 15.70% of the variance was attributable to sampling variance at Level 1, 4.35% to within-study variance at Level 2, and 79.95% to between-study variance at Level 3. Thus, only a small proportion of the total variance was located at the within-study level.

The full three-level model was subsequently compared with reduced models. Constraining the Level 2 variance component to zero did not significantly worsen model fit, *LRT* = 0.1007, *p* = 0.751. This indicates that the additional within-study variance component was not statistically supported. Constraining the Level 3 variance component to zero also did not reach conventional statistical significance, although the result approached the threshold, *LRT* = 3.7449, *p* = 0.053 > 0.05. As likelihood-ratio tests demonstrated that the full three-level model did not yield a statistically significant improvement relative to the corresponding reduced two-level models, the more parsimonious two-level meta-analytic model was adopted. This decision was intended to avoid estimating unnecessary variance components and reduce the risk of overfitting (Assink and Wibbelink, 2016).

### 5.2 Effect size and homogeneity tests

The 56 effect sizes of GenAI on learning motivation showed heterogeneity: *Q*_*motivation*_ [55] = 866.132, *p* < 0.001, *I*^2^ = 93.7%, *Tau*^2^ = 0.515 (see Table 2). This suggests true differences between studies, rather than random error, drive much of the variability in results. Therefore, we used a random-effects model (Higgins and Thompson, 2002). As *Hedge's g* = 0.764, 95% *CI* = [0.566, 0.962], 95% *PI* = [−0.689, 2.217]; *z* = 7.571, *p* < 0.001, students using GenAI showed more learning motivation than other students. However, due to the extremely high heterogeneity, the pooled effect should be interpreted as a positive average effect rather than evidence of a stable effect across all educational contexts.

**Table 2:** Random-effects model of the overall effect of GenAI on students' learning motivation.

*k*	*g*	95% *CI*	95% *PI*	*z*	Tau-squared			Heterogeneity test		
					*Tau* ^2^	*SE*	*Tau*	*Q*	*p*	*I* ^2^
56	0.764	[0.566, 0.962]	[−0.689, 2.217]	7.571^***^	0.515	0.134	0.718	866.132	< 0.001	0.937

*k*, number of effect sizes; Hedges's *g*, standardized mean difference corrected for small-sample bias; CI, confidence interval; PI, prediction interval; τ^2^, estimated between-effect variance; SE(τ^2^), standard error of τ^2^; τ, square root of τ^2^; *Q*, Cochran's heterogeneity statistic; *I*^2^, percentage of variability attributable to true heterogeneity. Positive values indicate higher learning motivation in the GenAI group than in the control group. ^***^p < 0.001. The same below.

### 5.3 Sensitivity analysis

To evaluate whether any individual effect sizes unduly influenced the meta-analytic results, we calculated studentized residuals (rstudent) for all 56 effect sizes. The residuals ranged from −1.3725 to 0.8925, all studentized residuals were below the outlier threshold of 2 (Viechtbauer, 2010), with most values clustered near zero, indicating no extreme deviations from the fitted model.

In addition, a leave-one-out sensitivity analysis was conducted by sequentially removing one effect size at a time and recalculating the pooled effect. The resulting effect sizes varied between 0.695 and 0.840, remaining well within the 95% confidence interval of the primary analysis (0.566 to 0.962), and all remained statistically significant (*p* ≤ 0.001). These results demonstrate that the main effect is robust and not driven by any single study or effect size.

### 5.4 Publication bias assessment

We tested for publication bias with a funnel plot and Egger et al.'s (1997) linear regression. Each of the following indicates high risk of publication bias: (a) many effect sizes asymmetrically arranged around the axis, especially outside the funnel, and (b) Egger's linear regression intercept is significant and far from zero.

The funnel plot showed no substantial asymmetries (see Figure 2). Egger's regression intercept did not reach statistical significance at the conventional 0.05 level, although the result was close to the threshold, *b* = 2.727, *t*_(54)_ = 1.945, *p* = 0.057 > 0.05. Therefore, the results provide no clear statistical evidence of publication bias.

**Figure 2:**
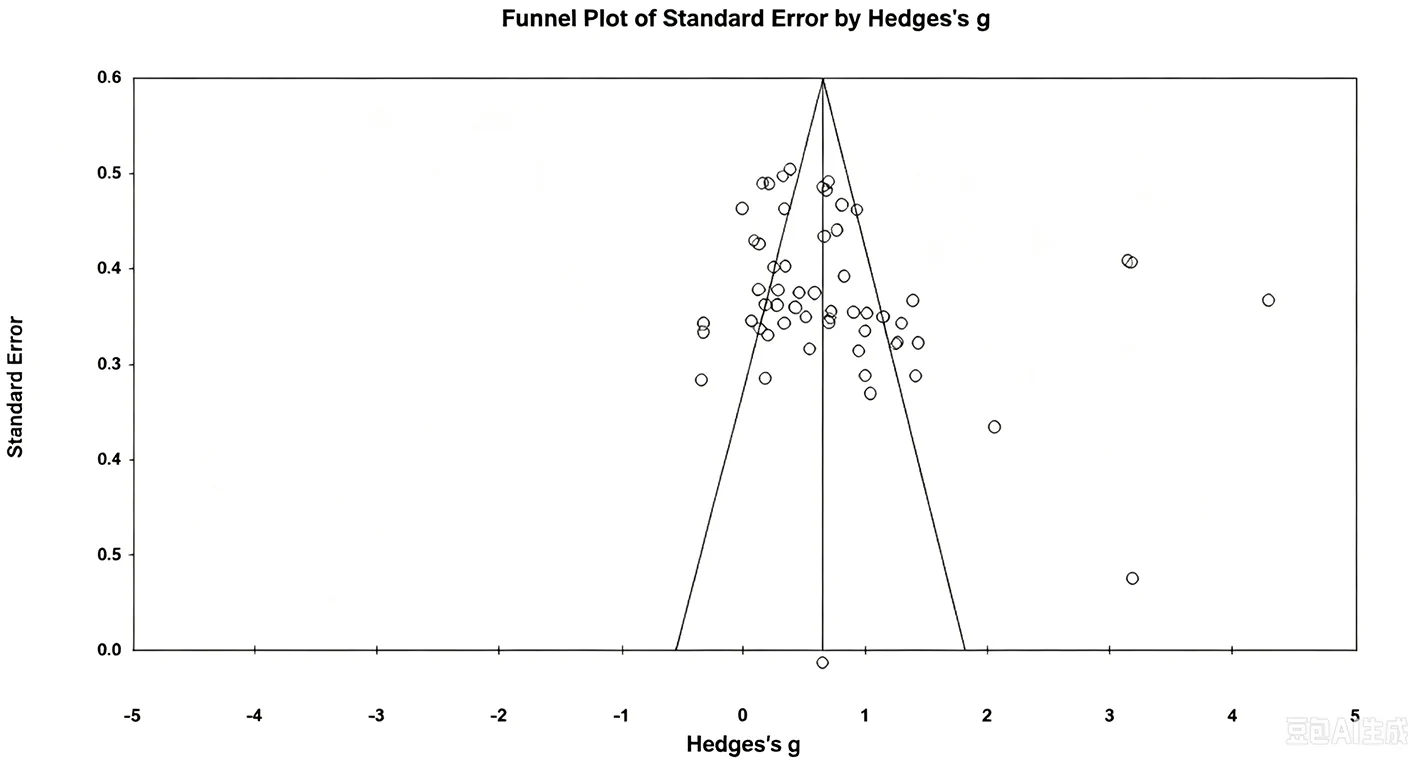
Funnel plot of the 56 effect sizes from 42 studies in the meta-analysis.

### 5.5 Moderator analysis

Given the very high heterogeneity and the possibility that study-level characteristics may be confounded, moderator analyses were treated as exploratory. These analyses were intended to identify possible sources of variation across effect sizes rather than to establish causal moderation. See subgroup analysis results for categorical moderators in Table 3 and meta-regression results for continuous variables in Table 4.

**Table 3:** The impact of using GenAI on learning motivation: moderating variables (categorical variables).

Moderator variables	*Q_*BET*_*	*k*	*g*	*SE*	95% *CI* for *g*	*Q_*W*_*	*I^2^*
**Type of motivation**	1.415 (*p* = 0.234)						
Extrinsic motivation		8	0.382	0.122	[0.143, 0.621]	22.130^**^	0.684
Intrinsic motivation		36	0.549	0.070	[0.412, 0.686]	154.936^***^	0.774
**Culture**	7.594^**^ (*p* = 0.006)						
Collectivism		49	0.815	0.118	[0.585, 1.046]	769.721^***^	0.938
Individualism		7	0.367	0.112	[0.147, 0.587]	27.919^***^	0.785
**Education stage**	13.536^**^ (*p* = 0.001)						
Primary School		4	0.311	0.119	[0.077, 0.544]	1.948	0.000
Secondary School		9	0.360	0.104	[0.157, 0.563]	25.810^**^	0.690
University		43	0.886	0.127	[0.637, 1.136]	778.346^***^	0.946
**GenAI function**	0.011 (*p* = 0.916)						
Intelligent tutoring systems		30	0.759	0.114	[0.536, 0.982]	295.243^***^	0.902
Personalized learning support		25	0.782	0.191	[0.407, 1.157]	560.917^***^	0.957
**GenAI device type**	11.913^**^ (*p* = 0.001)						
Mobile device		15	0.782	0.120	[0.545, 1.018]	68.070^***^	0.794
Personal computer		19	0.303	0.068	[0.169, 0.438]	47.014^***^	0.617
**Publication year**	3.519 (*p* = 0.172)						
2023		9	0.644	0.175	[0.300, 0.988]	33.804^***^	0.763
2024		19	0.552	0.099	[0.358, 0.745]	93.616^***^	0.808
2025		28	0.929	0.176	[0.585, 1.273]	693.288^***^	0.961
**Subject domain**	23.785^***^ (*p* = 0.000)						
Information		8	0.291	0.125	[0.047, 0.535]	19.490^**^	0.641
Language		32	0.946	0.161	[0.630, 1.262]	738.283^***^	0.958
Math		4	0.711	0.181	[0.356, 1.066]	8.422^*^	0.644
Pedagogy		6	0.697	0.222	[0.261, 1.133]	30.635^***^	0.837
Science		3	0.109	0.110	[−0.107, 0.325]	0.137	0.000
**Intervention duration**	1.475 (*p* = 0.478)						
Long		13	0.456	0.095	[0.270, 0.642]	59.694^***^	0.799
Medium		17	0.643	0.122	[0.404, 0.883]	80.738^***^	0.802
Short		17	0.519	0.104	[0.315, 0.722]	64.178^***^	0.751

*Q*_*BET*_, between-subgroup heterogeneity statistic; *Q*_*W*_, Heterogeneity test with each group; *I*^2^, percentage of variability attributable to true heterogeneity within each subgroup.

**Table 4:** Univariate regression analysis of continuous variables (the proportion of female participants).

Parameter	Estimate	*SE*	*z*	*p*	95% *CI* for *b*	*Q_*E*_*	*I* ^2^
Learning motivation							
**Female**	***Q**_***Model***_***(1**, ***k*** **=** **25)** **=** **0.25**, ***p*** **=** **0.614**						
β_0_	0.434	0.276	1.570	0.116	[−0.106, 0.975]	105.640^***^	0.782
β_1_	0.266	0.527	0.500	0.614	[−0.768, 1.300]		

The moderator was coded as the proportion of female participants in the experimental group. β_0_, intercept; β_1_, meta-regression coefficient for the proportion of female participants; *Q*_*Model*_, test of the meta-regression model; *Q*_*E*_, residual heterogeneity statistic; *I*^2^, percentage of residual variability attributable to true heterogeneity.

#### 5.5.1 Demographics

Observed between-subgroup differences were found across cultural contexts (*Q*_*BET*_ = 7.594, *df* = 1, *p* < 0.05). This effect was larger in countries with predominantly collectivist cultures (*g* = 0.815, 95% *CI* = [0.585, 1.046]) than those with individualistic ones (*g* = 0.367, 95% *CI* = [0.147, 0.587]).

Meta-regression showed no significant association between gender and GenAI's effect on learning motivation (*Q*_Model(1, k = 25)_ = 0.25, *p* > 0.05; see Table 4).

Observed between-subgroup differences by education stage were found (*Q*_*BET*_ = 13.536, *df* = 2, *p* < 0.05). The effect was stronger among university students (*g* = 0.886, 95% *CI* = [0.637, 1.136]), followed by secondary school students (*g* = 0.360, 95% *CI* = [0.157, 0.563]), and finally primary school students (*g* = 0.311, 95% *CI* = [0.077, 0.544]).

#### 5.5.2 Characteristics of GenAI

Between-subgroup differences were not significant across GenAI function (*Q*_*BET*_ = 0.011, *df* = 1, *p* > 0.05).

Observed between-subgroup differences were found across GenAI device type (*Q*_*BET*_ = 11.913, *df* = 1, *p* < 0.05). Using GenAI on mobile devices (*g* = 0.782, 95% *CI* = [0.545, 1.018]) had a stronger effect on learning motivation than using it on computers (*g* = 0.303, 95% *CI* = [0.169, 0.438]).

#### 5.5.3 Study characteristics

Observed between-subgroup differences across subject domains were found (*Q*_*BET*_ = 23.785, *df* = 4, *p* < 0.05), with language-related studies showing the largest pooled effect (*g* = 0.946, 95% *CI* = [0.630, 1.262]), followed by mathematics (*g* = 0.711, 95% *CI* = [0.356, 1.066]) and pedagogy (*g* = 0.697, 95% *CI* = [0.261, 1.133]), then information (*g* = 0.291, 95% *CI* = [0.047, 0.535]), and lowest in science (*g* = 0.109, 95% *CI* = [−0.107, 0.325]).

Between-subgroup differences were not significant across intervention duration (*Q*_*BET*_ = 1.475, *df* = 2, *p* > 0.05) and type of motivation (*Q*_*BET*_ = 1.415, *df* = 1, *p* > 0.05).

Meta-regression showed no significant association between publication year and GenAI's effect on learning motivation (*Q*_*BET*_ = 3.519, *df* = 2, *p* > 0.05).

## 6 Discussion

This meta-analysis examined whether students who use GenAI exhibit greater learning motivation compared to those who do not. Due to the high heterogeneity among studies, a random effects model was adopted. Additionally, this study analyzed the influence of nine moderator variables on the effectiveness of GenAI.

### 6.1 The effect of GenAI on students' learning motivation

This meta-analysis demonstrated that, compared with conventional teaching conditions, GenAI produced a statistically significant improvement in students' overall learning motivation (*g* = 0.764), with significant positive effects detected for both intrinsic motivation and extrinsic motivation. Taken together, the synthesized evidence indicates that GenAI yields a moderate-to-large average effect on student motivation. Nevertheless, the extremely high between-study heterogeneity (*I*^2^ = 93.7%) indicates that the magnitude of this effect varies dramatically across study contexts. Consequently, our findings do not support the conclusion that GenAI reliably increases motivation across all educational environments.

This exploratory finding may be interpreted through the lens of self-determination theory (SDT; Ryan and Deci, 2020), which posits that motivation is enhanced when learning activities satisfy learners' basic psychological needs for autonomy, competence, and relatedness. In the context of the present findings, GenAI may help support these needs through features such as personalized tutoring, immediate feedback, and supportive interaction. With respect to autonomy, GenAI may help structure individualized learning pathways that could allow students to select content and regulate pacing according to their progress and interests (Sok and Heng, 2024). Its web-search and hyperlink functions may also enable learners to verify information and retain greater control over their information seeking (Cai and Tian, 2025). Regarding competence, GenAI may be able to diagnose learning status and adjust task difficulty dynamically (Adeshola and Adepoju, 2024), potentially aligning tasks more closely with learners' zones of proximal development and reducing frustration associated with overly challenging content. It may also provide scaffolding and opportunities for learners to practice and demonstrate skills, which could in turn strengthen perceived competence (Haraldsrud and Odden, 2025). In terms of relatedness, GenAI may respond promptly to learners' questions, support customizable conversational styles, and deliver targeted encouragement (Taani and Alabidi, 2025). By creating what may be a low-threat practice environment that could reduce avoidance behaviors (Ibrahim, 2025), GenAI may foster safe and enjoyable human–AI interaction experiences that could partially satisfy relatedness needs. Overall, to the extent that these psychological needs underpin motivational processes, these patterns suggest that GenAI may be more likely to foster improvements in students' learning motivation relative to conventional instruction. In addition, GenAI's interactivity and perceived novelty may enhance learning enjoyment and could stimulate learners' curiosity and willingness to explore (Acquah et al., 2025). Its multimodal content-generation capabilities may also provide rich multimedia elements such as audio and images, which may help capture attention more effectively (Chen et al., 2025) and reduce cognitive load (Ji et al., 2025), potentially making knowledge processing and retention less cognitively demanding. Taken together, these proposed mechanisms may operate in combination to contribute to overall improvements in students' learning motivation.

### 6.2 Moderators of the effect of GenAI on students' learning motivation

The significant moderator findings should be interpreted cautiously. Although culture, education stage, subject domain, and device type were statistically associated with differences in average effect sizes, these variables were study-level characteristics rather than experimentally manipulated conditions. Therefore, the subgroup results are best understood as exploratory signals of possible boundary conditions. They may help explain why GenAI effects differed across studies, but they do not establish that culture, device type, subject domain, or education stage causally determined the motivational effects of GenAI.

#### 6.2.1 Type of motivation

Motivation type was not a significant moderator. To test H-1a and H-1b, subgroup analyses were conducted for intrinsic and extrinsic motivation. GenAI showed positive effects on both intrinsic and extrinsic motivation. Therefore, H-1a and H-1b were supported. However, the between-group comparison between intrinsic and extrinsic motivation was not statistically significant. Therefore, H-1c was not supported. Although self-determination theory distinguishes intrinsic motivation from extrinsic motivation, the present findings did not show a reliable difference between GenAI's effects on these two forms of motivation. This result suggests that GenAI may influence both internally driven engagement and externally oriented learning goals, but the current evidence does not justify the claim that its effect is reliably stronger for one type of motivation than the other. Overall, these non-significant moderator findings help define the boundary of the present conclusions and indicate that the significant moderator results should be interpreted selectively and cautiously.

#### 6.2.2 Culture

Our exploratory findings suggest that cultural context may contribute to heterogeneity in GenAI's effects on learning motivation, with stronger effects in collectivist societies. Collectivist cultural contexts tend to emphasize group honor and extrinsic rewards associated with academic success (Zhou et al., 2020). This cultural orientation may lead students to view GenAI as a practical tool for improving academic performance and meeting the expectations of teachers and parents, which could in turn encourage greater engagement with GenAI tools and may help strengthen extrinsic motivation. This tentative interpretation aligns with prior cross-cultural evidence showing that students in Hong Kong were more likely to prioritize academic competition and performance as core motivators, whereas students in the United Kingdom were more driven by personal interests (Lo, 2024). On this basis, when GenAI is perceived to support improved performance, its motivational benefits may be more pronounced in collectivist settings. By contrast, learners in individualist cultures tend to place greater value on autonomy and self-actualization. Their adoption of external learning tools tends to be shaped more by individual utility judgments than by social conformity or normative expectations (Oyserman et al., 2002; Srite and Karahanna, 2006). To the extent that GenAI is perceived as intrusive or undermining of learner autonomy, its potential motivational benefits may be attenuated in these contexts.

#### 6.2.3 Education stage

Our exploratory subgroup analysis suggests that student education level may moderate the effects of GenAI on learning motivation, with university students appearing to show larger motivational gains than secondary school students, who in turn appeared to benefit more than primary school students. Relative to younger learners, older learners tend to exhibit stronger learning skills and cognitive capacities, including abstract thinking (Piaget, 2016), metacognitive strategies (Akturk and Sahin, 2011), self-directed learning capacity (Sbaih et al., 2024), self-regulatory competence (Zimmerman, 2002), and higher technology acceptance (Zheng et al., 2017). These capabilities may support more strategic use of GenAI for knowledge extension and problem solving (Al-Abdullatif and Alsubaie, 2024; Teng, 2025), which could potentially contribute to greater feelings of accomplishment and satisfaction, and may subsequently help strengthen learning motivation. In contrast, primary school students generally have shorter sustained attention spans and weaker inhibitory control for maintaining goal-directed focus (Rothbart and Posner, 2007), and children aged 5–12 have been found to show greater attentional declines and task switching under high task load (Betts et al., 2006). Given that GenAI often generates multiple response paths and substantial textual output (Law, 2024), younger learners may be more susceptible to salient but task-irrelevant content, which could lead to cognitive overload. Taken together, these developmental differences may partially offset GenAI's potential motivational benefits among primary school students, which could help explain the smallest positive effect observed at this educational stage.

#### 6.2.4 GenAI function

GenAI function was not a significant moderator. Although assessment tools, intelligent tutoring systems, and personalized learning support may differ conceptually, their actual classroom implementations often overlap. For example, an AI tutor may simultaneously provide feedback, personalization, and assessment-related guidance. The non-significant result may therefore reflect the broadness of the current functional categories rather than the absence of meaningful differences among GenAI applications.

#### 6.2.5 GenAI device type

Exploratory analysis suggests that GenAI device type may be a potential moderator of GenAI's effects on learning motivation, a pattern that could plausibly be explained by the greater portability and accessibility of mobile devices. Mobile learning is broadly characterized by contextualized, personalized, and convenient access, which may support self-directed learning across time and place (Sharples et al., 2010). In practical learning scenarios, when learners encounter difficulties, they could readily access GenAI support via smartphones; such timely access may help resolve confusion and sustain learning momentum (Zhou et al., 2024). This ubiquitous, on-demand access may also help strengthen learners' perceived autonomy, lower barriers to tool use, and reduce perceived learning costs (Demir and Akpinar, 2018). As operational demands associated with accessing GenAI are reduced, learners might be able to allocate more cognitive resources to core learning tasks and AI-mediated interactions, potentially fostering more enjoyable and immersive learning experiences. Consistent with the Technology Acceptance Model, perceived usefulness and ease of use remain central determinants of technology attitudes and behavioral intentions (Davis and Granić, 2024). Given that smartphones and tablets are easier to operate and provide more intuitive interfaces, learners may experience smoother interactions and more favorable feedback; these exploratory patterns tentatively point to relatively stronger usage intentions and intrinsic motivation compared with desktop-based use, which is often more constrained by location and procedure.

#### 6.2.6 Publication year

Hypothesis 2, which posited that the link between GenAI and learning motivation would be stronger in studies published in later years, was not supported by the meta-analytic results. Publication year was not a significant moderator. Although GenAI technologies have developed rapidly, the studies included in this meta-analysis were concentrated within a short time window from 2023 to 2025. This compressed period may limit the ability to detect reliable year-to-year changes in motivational effects. Therefore, the absence of a significant publication-year effect should be interpreted cautiously rather than as evidence that technological development is irrelevant to GenAI's educational impact.

#### 6.2.7 Subject domain

The exploratory subject-domain finding suggested that average effects were largest in language-related courses. GenAI may improve English writing performance and writing motivation (Liu et al., 2024), support reading comprehension and cognitive load management (Yuan, 2025), and reduce speaking anxiety, thereby encouraging more active oral practice (Li and You, 2025). From a second language acquisition perspective, according to Input Hypothesis, GenAI can provide comprehensible input individualized tasks, and one-to-one interactive support, thereby increasing opportunities for practice and sustaining motivation (Li et al., 2024; Krashen, 1985). In addition, its immediate corrective feedback also aligns with the Interaction Hypothesis by supporting attention to form and meaning negotiation (Long, 1996). By delivering immediate corrective feedback and explanations on learners' output, including pronunciation and grammar, GenAI may refine linguistic structures and foster a more supportive psychological climate for learning (Wu et al., 2024), thus reinforcing motivation. In contrast, motivational benefits were smaller in information technology and science, aligning with prior meta-analytic conclusions (Ma and Zhong, 2025). In information-related subjects, students' earlier and more frequent exposure to digital tools may weaken GenAI's novelty value and attenuate novelty-driven motivational gains (Singh et al., 2023). In science education where hands-on practice and inquiry are foundational (Furtak and Penuel, 2019), dialogic support from tools such as ChatGPT is not able to fully replace authentic experimentation and operational experience, likely constraining its motivational impact. Notably, these subgroup findings should be interpreted with caution. Owing to the uneven distribution of included studies across subject domains, several subgroups contained relatively small sample sizes. The observed differences in motivational effect sizes may be partially attributed to imbalanced sample distribution, rather than representing a robust, genuine moderating effect of subject domain.

#### 6.2.8 Intervention duration

Intervention duration did not significantly moderate the effect. This suggests that the length of exposure alone may not determine whether GenAI supports students' motivation. Shorter interventions may benefit from novelty and immediate feedback, whereas longer interventions may require sustained instructional design, teacher scaffolding, and meaningful integration into learning activities to maintain motivational benefits. Thus, future research should examine not only intervention duration but also the quality and intensity of GenAI-supported learning activities.

#### 6.2.9 Gender

Hypothesis 3 was not supported by the present meta-analytic findings. Gender did not significantly moderate the effect of GenAI on learning motivation. Inconsistent with previous studies, the present meta-analysis did not provide reliable evidence that GenAI produces stronger motivational benefits for male or female students. This result may indicate that motivational responses to GenAI are shaped less by gender alone than by more proximal factors, such as task design, teacher guidance, AI literacy, and learners' prior experience with digital tools.

### 6.3 Implications

This study suggests a positive effect of GenAI on learning motivation with observed between-subgroup differences were found across cultural contexts, education stage, GenAI device type and subject domains. However, these findings should be treated as exploratory because of the uneven sample-size distribution across some subgroups and the substantial heterogeneity.

Specifically, classroom-based use of GenAI appeared to offer instructional benefits, as its immediate feedback and personalized guidance may foster more adaptive learning motivation. This pattern is broadly consistent with prior meta-analytic evidence indicating that GenAI can improve learning outcomes (Liu X. et al., 2025) and support the cultivation of higher-order thinking (Nie et al., 2025). In turn, these results align with the proposition that more positive learning motivation may contribute to better academic performance (Alhadi and Saputra, 2017). Future meta-analyses could examine these pathways as potential mediating mechanisms.

At the same time, cultural context may influence GenAI's effectiveness, with exploratory findings suggesting potentially larger effects in countries where collectivist values predominate. University instructors might consider incorporating GenAI to leverage these benefits, whereas primary school teachers may need to exercise caution to prevent students from becoming overly absorbed in entertaining interactions at the expense of learning objectives. Regarding subject domains, language teachers may explore using GenAI to correct pronunciation, support fluency in speaking, and provide guidance for writing, though these findings remain exploratory and context-dependent.

## 7 Limitations and future research

Several limitations should be considered when interpreting the findings of this meta-analysis. First, heterogeneity across studies was substantial. Although a random-effects model was used and moderator analyses were conducted to explore possible sources of variation, the large heterogeneity indicates that the pooled effect should be interpreted as an average estimate rather than as evidence of a uniform effect across educational contexts. Differences in learners, instructional designs, GenAI tools, subject domains, cultural settings, and implementation conditions may have contributed to the variability in effects. Future meta-analyses should continue to examine boundary conditions and, where sufficient evidence is available, use more fine-grained subgroup analyses or meta-regression models to explain this heterogeneity.

Second, potential effect-size dependence remains a limitation. Some primary studies contributed more than one effect size because they reported multiple motivation outcomes, intervention conditions, or moderator-relevant comparisons. Although a supplementary three-level meta-analysis suggested that within-study variance was small and did not materially alter the main findings, some dependency may remain because effect sizes from the same study can share participants, measures, or intervention features. Therefore, the pooled effect and moderator findings should be interpreted cautiously. Future meta-analyses with a larger evidence base should compare study-level aggregation, multilevel meta-analysis, and robust variance estimation to further examine the influence of effect-size dependence.

Third, some moderator groups were unevenly distributed, which may reduce statistical power, produce unstable subgroup estimates, and confound moderator findings with country, sample, design, or measurement characteristics. Therefore, the moderator findings should be interpreted as exploratory and context-sensitive.

Fourth, measurement variation across primary studies may have influenced the findings. Although all studies measured learning motivation quantitatively, they used different instruments and operational definitions. Some studies reported intrinsic or extrinsic motivation separately, whereas others reported general, total, or combined learning motivation scores. Such variation may have introduced construct heterogeneity and partly contributed to differences in effect sizes.

Fifth, this meta-analysis included only studies published in English. As GenAI is being adopted globally, relevant studies published in other languages may have been missed. Future reviews could include multilingual searches to provide a more comprehensive evidence base.

Sixth, the present meta-analysis was limited in sample coverage. The included studies focused on students from primary school through university and did not include kindergarten-aged learners or students receiving special education services, such as learners with autism or physical disabilities. These omissions may constrain the generalizability of the findings.

Seventh, moderators were tested independently, and we did not examine potential interactions among moderators. Future research should explore whether and how variables such as educational stage, subject domain, cultural context, device type, and GenAI function interact to shape GenAI's motivational effects.

Future meta-analyses should continue to assess publication bias using multiple complementary approaches as the evidence base expands.

## 8 Conclusion

This random-effects meta-analysis synthesized 56 effect sizes from 42 studies involving 6,059 participants to examine the association between GenAI-supported learning and students' learning motivation. Pooled results indicated that GenAI use was associated with higher learning motivation on average, yet the very high between-study heterogeneity suggests that the magnitude and consistency of this effect vary substantially across study contexts. Exploratory moderation analyses identified tentative patterns of subgroup differences. Specifically, the positive association between GenAI and learning motivation appeared larger for college students relative to secondary and elementary school students, greater in collectivistic cultures compared to individualistic cultures, more prominent when GenAI was delivered via mobile devices rather than personal computers, and largest among studies in language disciplines. In contrast, no statistically significant moderation effects were detected for gender, publication year, intervention duration, GenAI function, or motivation type in the current analyses. Given the very high overall heterogeneity, imbalanced study distribution across subgroups, and potential confounding at the study level, these subgroup findings should be interpreted as preliminary exploratory patterns rather than confirmed moderating effects.

## Data Availability

The original contributions presented in the study are included in the article/supplementary material, further inquiries can be directed to the corresponding author.
