# Supplementary material for: Effects of generative artificial intelligence on students' learning motivation: evidence from a meta-analysis
Source: Front Psychol. 2026 Sep 24;17:1832451. doi: 10.3389/fpsyg.2026.1832451 (PMC13647323; doi:10.3389/fpsyg.2026.1832451)
Supplement: Supplementary file 1 [file Supplementary_file_1.docx]

**Appendix A**

**Table 5**

*Database-specific search strategies*

| **Database** | **Search date** | **Search fields** | **Complete search strategy** | **Filters applied** |
| --- | --- | --- | --- | --- |
| **Web of Science** | November, 2025 | Topic (including Title, Abstract, Author Keywords, and Keywords Plus) | TS = (("generative artificial intelligence" OR "generative AI" OR GenAI OR ChatGPT OR GPT OR "large language model*" OR LLM* OR "AI chatbot*" OR "AI tutor*" OR "AI tutoring" OR "artificial intelligence assistant" OR "AI-powered") AND ("learning motivation" OR "academic motivation" OR "motivation to learn" OR "intrinsic motivation" OR "extrinsic motivation")) | Language: English; Publication years: 2023–2025; Document types: Article OR Proceedings Paper |
| **Scopus** | November, 2025 | Title, Abstract, and Keywords | TITLE-ABS-KEY(("generative artificial intelligence" OR "generative AI" OR GenAI OR ChatGPT OR GPT OR "large language model" OR LLM OR "AI chatbot" OR "AI tutor*" OR "AI tutoring" OR "artificial intelligence assistant*" OR "AI-powered") AND ("learning motivation" OR "academic motivation" OR "motivation to learn" OR "intrinsic motivation" OR "extrinsic motivation")) | Language: English; Publication years: 2023–2025; Document types: Article OR Conference Paper |
| **ERIC** | November, 2025 | Title, Abstract, and Descriptor | ("generative artificial intelligence" OR "generative AI" OR GenAI OR ChatGPT OR "large language model*" OR "AI chatbot*" OR "AI tutor*" OR "AI tutoring") AND ("learning motivation" OR "academic motivation" OR "motivation to learn" OR motivation) | Language: English; Publication years: 2023–2025; Peer-reviewed publications |
| **EBSCO** | November, 2025 | Title (TI), Abstract (AB), Subject Terms (SU) | ((TI ("generative artificial intelligence" OR "generative AI" OR GenAI OR ChatGPT OR "large language model*" OR "AI chatbot*" OR "AI tutor") OR AB ("generative artificial intelligence" OR "generative AI" OR GenAI OR ChatGPT OR "large language model*" OR "AI chatbot*" OR "AI tutor")) AND (TI ("learning motivation" OR "academic motivation" OR "motivation to learn" OR motivation) OR AB ("learning motivation" OR "academic motivation" OR "motivation to learn" OR motivation))) | Language: English; Publication years: 2023–2025; Scholarly (Peer Reviewed) Journals |
| **ProQuest** | November, 2025 | Title, Abstract, Subject Terms | ("generative artificial intelligence" OR "generative AI" OR GenAI OR ChatGPT OR "large language model*" OR "AI chatbot" OR "AI tutor" OR "AI tutoring") AND ("learning motivation" OR "academic motivation" OR "motivation to learn" OR motivation) | Language: English; Publication years: 2023–2025; Peer-reviewed publications |

**Table 6**

*BQAPS Scores for the 42 Included Studies*

| **Questions** | **Q1** | **Q2** | **Q3** | **Q4** | **Q5** | **Q6** | **Q7** | **Q8** | **Q9** | **Q10** | **Q11** | **Q12** |  |
| --- | --- | --- | --- | --- | --- | --- | --- | --- | --- | --- | --- | --- | --- |
| **ITEM** | 1 | 2 | 3 | 4 | 5 | 6 | 7 | 8 | 9 | 10 | 11 | 12 | **Total score** |
| Ahmad (2024) | 2 | 2 | 1 | 2 | 2 | 2 | 2 | 2 | 1 | 1 | 2 | 1 | 20 |
| Azamatova et al. (2023) | 2 | 2 | 1 | 2 | 2 | 2 | 2 | 2 | 1 | 1 | 2 | 1 | 20 |
| Beketov et al. (2024) | 2 | 2 | 1 | 2 | 2 | 2 | 2 | 2 | 1 | 1 | 2 | 1 | 20 |
| Chen et al. (2024) | 2 | 2 | 1 | 2 | 2 | 2 | 2 | 2 | 2 | 1 | 2 | 2 | 22 |
| Chen et al. (2025)a | 2 | 2 | 1 | 2 | 2 | 2 | 2 | 2 | 1 | 1 | 2 | 1 | 20 |
| Chen et al. (2025)b | 2 | 2 | 1 | 2 | 2 | 2 | 2 | 2 | 1 | 1 | 2 | 1 | 20 |
| Chen & Chang(2024) | 2 | 2 | 1 | 2 | 2 | 2 | 2 | 2 | 2 | 2 | 2 | 2 | 23 |
| Choi & Kim (2025) | 2 | 2 | 1 | 2 | 2 | 2 | 2 | 2 | 2 | 2 | 2 | 1 | 22 |
| Cubillos et al. (2025) | 2 | 2 | 1 | 2 | 2 | 2 | 2 | 2 | 2 | 1 | 2 | 1 | 21 |
| Dahri et al. (2025) | 1 | 2 | 1 | 2 | 2 | 2 | 2 | 2 | 2 | 2 | 2 | 1 | 21 |
| Dou & Sun (2025) | 1 | 2 | 1 | 2 | 2 | 2 | 2 | 2 | 2 | 2 | 2 | 1 | 21 |
| Fan et al. (2025)a | 2 | 2 | 1 | 2 | 2 | 2 | 2 | 2 | 2 | 2 | 2 | 1 | 22 |
| Fan et al. (2025)b | 2 | 2 | 1 | 2 | 2 | 2 | 2 | 2 | 2 | 1 | 2 | 1 | 21 |
| Huang & Mizumoto (2024) | 1 | 2 | 1 | 2 | 2 | 2 | 2 | 2 | 2 | 1 | 2 | 1 | 20 |
| Jafari et al. (2025) | 1 | 2 | 1 | 2 | 2 | 2 | 2 | 2 | 1 | 1 | 2 | 1 | 19 |
| Li et al. (2025) | 1 | 2 | 1 | 2 | 2 | 2 | 2 | 2 | 2 | 1 | 2 | 1 | 20 |
| Li (2023) | 1 | 2 | 1 | 2 | 2 | 2 | 2 | 2 | 2 | 1 | 2 | 1 | 20 |
| Liu et al. (2024)a | 1 | 2 | 1 | 2 | 2 | 2 | 2 | 2 | 2 | 1 | 2 | 1 | 20 |
| Liu et al. (2024)b | 2 | 2 | 1 | 2 | 2 | 2 | 2 | 2 | 2 | 1 | 2 | 1 | 21 |
| Liu (2025) | 2 | 2 | 1 | 2 | 2 | 2 | 2 | 2 | 2 | 1 | 2 | 1 | 21 |
| Meyer et al. (2024) | 2 | 2 | 1 | 2 | 2 | 2 | 2 | 2 | 2 | 1 | 2 | 1 | 21 |
| Mohammed & Khalid (2025) | 1 | 2 | 1 | 2 | 2 | 2 | 2 | 2 | 2 | 1 | 2 | 1 | 20 |
| Podoliak et al. (2025) | 2 | 2 | 1 | 2 | 2 | 2 | 2 | 2 | 2 | 1 | 2 | 1 | 21 |
| Se?ler et al. (2024) | 1 | 2 | 1 | 2 | 2 | 2 | 2 | 2 | 1 | 1 | 2 | 1 | 19 |
| Shao (2025) | 1 | 2 | 1 | 2 | 2 | 2 | 2 | 2 | 2 | 1 | 2 | 1 | 20 |
| Silitonga et al. (2023) | 1 | 2 | 1 | 2 | 2 | 2 | 2 | 2 | 2 | 1 | 2 | 1 | 20 |
| Song & Song (2023) | 2 | 2 | 1 | 2 | 2 | 2 | 2 | 2 | 2 | 1 | 2 | 1 | 21 |
| Suciati et al. (2024) | 1 | 2 | 1 | 2 | 2 | 2 | 2 | 1 | 2 | 1 | 2 | 1 | 19 |
| Teng (2024) | 1 | 2 | 1 | 2 | 2 | 2 | 2 | 2 | 2 | 1 | 2 | 1 | 20 |
| Wang et al. (2025)a | 1 | 2 | 1 | 2 | 2 | 2 | 2 | 2 | 2 | 2 | 2 | 1 | 21 |
| Wang et al. (2025)b | 1 | 2 | 1 | 2 | 2 | 1 | 2 | 2 | 2 | 1 | 2 | 1 | 19 |
| Wang et al. (2025)c | 1 | 2 | 1 | 2 | 2 | 2 | 2 | 2 | 2 | 2 | 1 | 1 | 20 |
| Wang et al. (2025)d | 2 | 2 | 1 | 2 | 2 | 2 | 2 | 2 | 2 | 2 | 2 | 1 | 22 |
| Wei, L. (2023) | 1 | 2 | 1 | 2 | 2 | 2 | 2 | 2 | 2 | 2 | 1 | 1 | 20 |
| Wu et al. (2024)1 | 1 | 2 | 1 | 2 | 2 | 2 | 2 | 2 | 1 | 1 | 1 | 1 | 18 |
| Y?ld?z (2023) | 1 | 2 | 1 | 2 | 2 | 1 | 2 | 2 | 2 | 1 | 1 | 1 | 18 |
| Yilmaz & Yilmaz (2023) | 2 | 2 | 1 | 2 | 2 | 1 | 2 | 2 | 2 | 2 | 2 | 1 | 21 |
| Yuan & Liu (2025) | 2 | 2 | 1 | 2 | 2 | 1 | 2 | 2 | 2 | 2 | 2 | 1 | 21 |
| Zhang et al. (2025)a | 2 | 2 | 1 | 2 | 2 | 2 | 2 | 2 | 2 | 2 | 2 | 1 | 22 |
| Zhang et al. (2025)b | 1 | 2 | 1 | 2 | 2 | 2 | 2 | 2 | 2 | 2 | 2 | 1 | 21 |
| Zhang (2024) | 1 | 2 | 1 | 2 | 2 | 2 | 2 | 2 | 1 | 2 | 2 | 1 | 20 |
| Zhou et al. (2025) | 1 | 2 | 1 | 2 | 2 | 1 | 2 | 2 | 2 | 1 | 1 | 1 | 18 |

*Note: Q1:* *Did it allocate participants randomly into experimental and control group in an experiment study? Or, Did it extract sample randomly in a correlation study?*

*Q2:* *Are other conditions same except for the treatment in the experimental group in an experiment study? Or, Are the conditions same in all sub-group in a correlation study?*

*Q3:* *Did the experimenters (or the investigators who distribute the quesationnaire) and participants know the purpose of the study?*

*Q4:* *Did it employ standerdized measuring tool in each primary study?*

*Q5:* *Did the experimental and control group use the same measuring tool in an experiment study? Or, Did each sub-group use the same measuring tool in a correlation study?*

*Q6:* *Did it record the loss of participants and explain the reasons? That is, Did it record the rate of participants who withdrawed from the experiment, or the number of missing in the correaltion study?*

*Q7:* *Did the primary study report results correctly?*

*Q8:* *Did the primary study report the characteristics of sample clearly?*

*Q9:* *Did it test the reliability of the measuring tools used in the primary studies?*

*Q10:* *Did it test the validity of the measuring tools used in the primary studies?*

*Q11:* *Did it employ reasonable statistical methods in the primary studies? the word reasonable here means that this method used can answer its research question and is suitable for the data.*

*Q12:* *Did it provide some evidence to support generalizing its results into other groups or population?*

**Table 7**

*Characteristics of the 42 Studies Included in the Meta-analysis*

| **Authors** | **Effect** | **Academic Subject** | **Motivation type** | **GenAI function** | **Culture** | **Year** | **Education stage** | **Gender** | **Intervention Duration** | **GenAI device** |
| --- | --- | --- | --- | --- | --- | --- | --- | --- | --- | --- |
| Ahmad (2024) | 0.190 | science | IM | ITS | Collect | 2024 | Primary | NA | medium | NA |
| Azamatova et al. (2023) | 1.420 | language | IM | PLS | Collect | 2023 | Univ. | 0.531 | medium | NA |
| Beketov et al. (2024) | 0.787 | medicine | NA | ITS | Collect | 2024 | Univ. | 0.496 | medium | NA |
| Chen et al. (2024)1 | 0.111 | language | IM | PLS | Collect | 2024 | Primary | 0.500 | short | PC |
| Chen et al. (2024)2 | 0.445 | language | EM | PLS | Collect | 2024 | Primary | 0.500 | short | PC |
| Chen et al. (2025)a | 0.694 | NA | IM | ITS | Collect | 2025 | Second. | 0.576 | medium | PC |
| Chen et al. (2025)b | 0.683 | art | IM | ITS | Collect | 2025 | Univ. | NA | short | PC |
| Chen & Chang(2024)1 | 0.113 | science | IM | ITS | Collect | 2024 | Second. | NA | short | PC |
| Chen & Chang(2024)2 | 0.073 | science | IM | ITS | Collect | 2024 | Second. | NA | short | PC |
| Choi & Kim (2025)1 | 0.327 | information | IM | PLS | Individ | 2025 | Second. | 0.472 | medium | PC |
| Choi & Kim (2025)2 | -0.027 | information | EM | PLS | Individ | 2025 | Second. | 0.472 | medium | PC |
| Cubillos et al. (2025) | -0.347 | information | IM | ITS | Collect | 2025 | Univ. | NA | NA | NA |
| Dahri et al. (2025) | 3.183 | pedagogy | NA | ITS | Collect | 2025 | Univ. | 0.625 | short | MD |
| Dou & Sun (2025) | 0.644 | language | IM | ITS | Collect | 2025 | Univ. | 0.769 | short | NA |
| Fan et al. (2025)a1 | 0.234 | information | IM | ITS | Collect | 2025 | Univ. | 0.368 | long | PC |
| Fan et al. (2025)a2 | 0.325 | information | IM | ITS | Collect | 2025 | Univ. | 0.333 | long | PC |
| Fan et al. (2025)b | 0.058 | language | IM | ITS | Collect | 2025 | Univ. | NA | short | PC |
| Huang & Mizumoto (2024)1 | 0.188 | language | IM | PLS | Individ | 2024 | Univ. | NA | long | PC |
| Huang & Mizumoto (2024)2 | 0.136 | language | EM | PLS | Individ | 2024 | Univ. | NA | long | PC |
| Jafari et al. (2025) | 1.022 | language | NA | PLS | Collect | 2025 | Univ. | NA | medium | NA |
| Li et al. (2025) | 0.676 | pedagogy | IM | PLS | Collect | 2025 | Univ. | NA | long | NA |
| Li (2023)1 | 0.568 | pedagogy | IM | PLS | Collect | 2023 | Univ. | 0.571 | short | NA |
| Li(2023)2 | 0.270 | pedagogy | EM | PLS | Collect | 2023 | Univ. | 0.571 | short | NA |
| Liu et al. (2024)a1 | 0.173 | pedagogy | IM | ITS | Collect | 2024 | Univ. | 0.486 | short | MD |
| Liu et al. (2024)a2 | 0.412 | pedagogy | EM | ITS | Collect | 2024 | Univ. | 0.486 | short | MD |
| Liu et al. (2024)b | 0.500 | language | IM | PLS | Collect | 2024 | Primary | 0.563 | medium | MD |
| Liu (2025)1 | 4.282 | language | NA | PLS | Collect | 2025 | Univ. | NA | NA | NA |
| Liu (2025)2 | 3.155 | language | NA | ITS | Collect | 2025 | Univ. | NA | NA | NA |
| Liu (2025)3 | 3.121 | language | NA | PLS | Collect | 2025 | Univ. | NA | NA | NA |
| Meyer et al. (2024) | 0.372 | language | IM | AST | Individ | 2024 | Second. | NA | short | PC |
| Mohammed & Khalid (2025) | 0.632 | language | NA | ITS | Collect | 2025 | Univ. | NA | long | NA |
| Podoliak et al. (2025) | 0.808 | language | IM | ITS | Individ | 2025 | Univ. | NA | medium | MD |
| Seßler et al. (2024) | 1.396 | math | IM | ITS | Individ | 2024 | Univ. | NA | short | PC |
| Shao (2025) | 0.746 | language | IM | ITS | Collect | 2025 | Univ. | 0.756 | long | MD |
| Silitonga et al. (2023)1 | 1.123 | language | IM | PLS | Collect | 2023 | Univ. | NA | NA | MD |
| Silitonga et al. (2023)2 | 0.992 | language | EM | PLS | Collect | 2023 | Univ. | NA | NA | MD |
| Song & Song (2023) | 0.527 | language | IM | ITS | Collect | 2023 | Univ. | NA | long | NA |
| Suciati et al. (2024)1 | 1.277 | language | IM | ITS | Collect | 2024 | Univ. | NA | medium | NA |
| Suciati et al. (2024)2 | 0.883 | language | EM | ITS | Collect | 2024 | Univ. | NA | medium | NA |
| Teng (2024) | 2.058 | language | IM | PLS | Collect | 2024 | Univ. | 0.542 | long | NA |
| Wang et al. (2025)a1 | 0.977 | language | IM | ITS | Collect | 2025 | Univ. | NA | NA | NA |
| Wang et al. (2025)a2 | 1.246 | language | IM | ITS | Collect | 2025 | Univ. | NA | NA | NA |
| Wang et al. (2025)b | 1.234 | language | IM | ITS | Collect | 2025 | Second. | NA | short | MD |
| Wang et al. (2025)c | 0.912 | language | IM | ITS | Collect | 2025 | Univ. | 0.000 | NA | MD |
| Wang et al. (2025)d1 | 0.124 | information | NA | PLS | Collect | 2025 | Univ. | NA | medium | PC |
| Wang et al. (2025)d2 | 0.925 | information | NA | PLS | Collect | 2025 | Univ. | NA | medium | PC |
| Wei, L. (2023) | 0.322 | language | IM | ITS | Collect | 2023 | Univ. | 0.567 | long | MD |
| Wu et al. (2024)1 | 0.702 | math | IM | PLS | Collect | 2024 | Second. | 0.400 | short | MD |
| Wu et al. (2024)2 | 0.261 | math | EM | PLS | Collect | 2024 | Second. | 0.400 | short | MD |
| Yıldız (2023) | -0.332 | language | IM | PLS | Collect | 2023 | Univ. | NA | long | PC |
| Yilmaz & Yilmaz (2023) | 0.978 | information | NA | PLS | Collect | 2023 | Univ. | NA | medium | PC |
| Yuan & Liu (2025) | 0.309 | language | NA | ITS | Collect | 2025 | Univ. | 0.706 | long | MD |
| Zhang et al. (2025)a | -0.333 | language | IM | PLS | Collect | 2025 | Univ. | NA | medium | NA |
| Zhang et al. (2025)b | 0.167 | language | IM | PLS | Collect | 2025 | Univ. | 0.333 | medium | NA |
| Zhang (2024) | 1.377 | language | NA | ITS | Collect | 2024 | Univ. | NA | medium | MD |
| Zhou et al. (2025) | 0.662 | math | IM | ITS | Collect | 2025 | Univ. | 0.490 | long | NA |

**Appendix B**

References marked with an asterisk indicate studies included in the meta-analysis.

*Ahmad, F. B. (2024). The Impact of the Use of Artificial Intelligence-Based Gamification on the Development of the Motivation of Students of the Basic Stage towards Education. *International Society for Technology, Education, and Science.*

*Azamatova, A., Bekeyeva, N., Zhaxylikova, K., Sarbassova, A., & Ilyassova, N. (2023). The Effect of Using Artificial Intelligence and Digital Learning Tools Based on Project-Based Learning Approach in Foreign Language Teaching on Students' Success and Motivation. *International Journal of Education in Mathematics, Science and Technology, 11*(6), 1458-1475. https://doi.org/10.46328/ijemst.3712

*Beketov, V., Lebedeva, M., & Taranova, M. (2024). The use of artificial intelligence in teaching medical students to increase motivation and reduce anxiety during academic practice. *Current Psychology, 43*(16), 14367-14377. https://doi.org/10.1007/s12144-023-05471-7

*Chen, C. H., & Chang, C. L. (2024). Effectiveness of AI-assisted game-based learning on science learning outcomes, intrinsic motivation, cognitive load, and learning behavior. *Education and Information Technologies, 29*(14), 18621-18642. https://doi.org/10.1007/s10639-024-12553-x

*Chen, J., Mokmin, N. A. M., & Qi, S. (2025a). Generative AI-powered arts-based learning in middle school history: Impact on achievement, motivation, and cognitive load. *The Journal of Educational Research*, 1-13. https://doi.org/10.1080/00220671.2025.2510395

*Chen, J., Mokmin, N. A. M., & Su, H. (2025b). Integrating generative artificial intelligence into design and art course: Effects on student achievement, motivation, and self-efficacy. *Innovations in Education and Teaching International*, 1-16. https://doi.org/10.1080/14703297.2025.2503857

*Chen, Y., Zhang, X., & Hu, L. (2024). A progressive prompt-based image-generative AI approach to promoting students’ achievement and perceptions in learning ancient Chinese poetry. *Educational Technology & Society, 27*(2), 284-305. https://doi.org/10.30191/ETS.202404_27(2).TP01

*Choi, S., & Kim, H. (2025). The impact of a large language model-based programming learning environment on students’ motivation and programming ability. *Education and Information Technologies, 30*(6), 8109-8138. https://doi.org/10.1007/s10639-024-13107-x

*Cubillos, C., Mellado, R., Cabrera-Paniagua, D., & Urra, E. (2025). Generative Artificial Intelligence in Computer Programming: Does it enhance learning, motivation, and the learning environment? *IEEE Access*. https://doi.org/10.1109/ACCESS.2025.3532883

*Dahri, N. A., Al-Rahmi, W. M., Alhashmi, K. A., & Bashir, F. (2025). Enhancing Mobile Learning with AI-Powered Chatbots: Investigating ChatGPT's Impact on Student Engagement and Academic Performance. *International Journal of Interactive Mobile Technologies, 19*(11). https://doi.org/10.3991/ijim.v19i11.54643

*Dou, W., & Sun, X. (2025). Artificial intelligence tools: Improvement of motivation, psychological well-being, and psychological capital of EFL learners: A self-determination theory perspective. *Learning and Motivation, 92*, 102169. https://doi.org/10.1016/j.lmot.2025.102169

*Fan, G., Liu, D., Zhang, R., & Pan, L. (2025a). The impact of AI-assisted pair programming on student motivation, programming anxiety, collaborative learning, and programming performance: a comparative study with traditional pair programming and individual approaches. *International Journal of STEM Education, 12*(1), 16. https://doi.org/10.1186/s40594-025-00537-3

*Fan, Y., Tang, L., Le, H., Shen, K., Tan, S., Zhao, Y., ... & Gašević, D. (2025b). Beware of metacognitive laziness: Effects of generative artificial intelligence on learning motivation, processes, and performance. *British Journal of Educational Technology, 56*(2), 489-530. https://doi.org/10.1111/bjet.13544

*Huang, J., & Mizumoto, A. (2024). Examining the effect of generative AI on students’ motivation and writing self-efficacy. *Digital Applied Linguistics, 1*, 102324-102324. https://doi.org/10.29140/dal.v1.102324

* Jafari, S., Fakhraee, L., & Teimourtash, M. (2025). The Impact of Artificial Intelligence (chat GPT) on Writing Accuracy of Iranian Intermediate EFL Learners and Their Level of Motivation. *Technology Assisted Language Education*, *3*(1), 90-109. https://doi.org/10.22126/tale.2025.11123.1063

*Li, H. (2023). Effects of a ChatGPT-based flipped learning guiding approach on learners’ courseware project performances and perceptions. *Australasian Journal of Educational Technology, 39*(5), 40-58. https://doi.org/10.14742/ajet.8923

*Li, Y., Sadiq, G., Qambar, G., & Zheng, P. (2025). The impact of students’ use of ChatGPT on their research skills: The mediating effects of autonomous motivation, engagement, and self-directed learning. *Education and Information Technologies, 30*(4), 4185-4216. https://doi.org/10.1007/s10639-024-12981-9

*Liu, C. C., Wang, H. J., Wang, D., Tu, Y. F., Hwang, G. J., & Wang, Y. (2024a). An interactive technological solution to foster preservice teachers’ theoretical knowledge and instructional design skills: A chatbot-based 5E learning approach. *Interactive Learning Environments, 32*(10), 6698-6716. https://doi.org/10.1080/10494820.2023.2277761

*Liu, L. (2025). Impact of AI gamification on EFL learning outcomes and nonlinear dynamic motivation: Comparing adaptive learning paths, conversational agents, and storytelling. *Education and Information Technologies, 30*(8), 11299-11338. https://doi.org/10.1007/s10639-024-13296-5

*Liu, Z. M., Hwang, G. J., Chen, C. Q., Chen, X. D., & Ye, X. D. (2024b). Integrating large language models into EFL writing instruction: effects on performance, self-regulated learning strategies, and motivation. *Computer Assisted Language Learning*, 1-25. https://doi.org/10.1080/09588221.2024.2389923

*Meyer, J., Jansen, T., Schiller, R., Liebenow, L. W., Steinbach, M., Horbach, A., & Fleckenstein, J. (2024). Using LLMs to bring evidence-based feedback into the classroom: AI-generated feedback increases secondary students’ text revision, motivation, and positive emotions. *Computers and Education: Artificial Intelligence, 6*, 100199. https://doi.org/10.1016/j.caeai.2023.100199

*Mohammed, S. J., & Khalid, M. W. (2025). Under the world of AI-generated feedback on writing: Mirroring motivation, foreign language peace of mind, trait emotional intelligence, and writing development. *Language Testing in Asia, 15*(1), 7. https://doi.org/10.1186/s40468-025-00343-2

* Podoliak, M., Zagranovska, O., Posmitna, V., Golovchak, N., & Kushnirchuk, O. (2025). Evaluating the impact of AI-based tools on language proficiency and motivation: Experimental evidence from philology students in Ukraine. *Journal of Research, Innovation and Technologies*, *4*(3), 271-282. https://doi.org/10.56578/jorit040303.

*Seßler, K., Kepir, O., & Kasneci, E. (2024, September). Enhancing Student Motivation Through LLM-Powered Learning Environments: A Comparative Study. In *European Conference on Technology Enhanced Learning* (pp. 156-162). Cham: Springer Nature Switzerland. https://doi.org/10.1007/978-3-031-72312-4_21

*Shao, S. (2025). The role of AI tools on EFL students’ motivation, self-efficacy, and anxiety: Through the lens of control-value theory. *Learning and Motivation, 91*, 102154. https://doi.org/10.1016/j.lmot.2025.102154

*Silitonga, L. M., Hawanti, S., Aziez, F., Furqon, M., Zain, D. S. M., Anjarani, S., & Wu, T. T. (2023, August). The impact of AI chatbot-based learning on students’ motivation in English writing classroom. In *International conference on innovative technologies and learning* (pp. 542-549). Cham: Springer Nature Switzerland. https://doi.org/10.1007/978-3-031-40113-8_53

*Song, C., & Song, Y. (2023). Enhancing academic writing skills and motivation: assessing the efficacy of ChatGPT in AI-assisted language learning for EFL students. *Frontiers in psychology, 14*, 1260843. https://doi.org/10.3389/fpsyg.2023.1260843

*Suciati, S., Silitonga, L. M., Wiyaka, Huang, C. Y., & Anggara, A. A. (2024, July). Enhancing engagement and motivation in English writing through AI: The impact of ChatGPT-supported collaborative learning. In *International Conference on Innovative Technologies and Learning* (pp. 205-214). Cham: Springer Nature Switzerland. https://doi.org/10.1007/978-3-031-65884-6_21

*Teng, M. F. (2024). “ChatGPT is the companion, not enemies”: EFL learners’ perceptions and experiences in using ChatGPT for feedback in writing. *Computers and Education: Artificial Intelligence, 7*, 100270. https://doi.org/10.1016/j.caeai.2024.100270

*Wang, C., Wang, B., & Xu, D. (2025a). The role of ChatGPT and grammarly in promoting emotion regulation, psychological well-being, motivation, and academic writing in Chinese college students: A self-determination theory perspective. *Learning and Motivation, 90*, 102131. https://doi.org/10.1016/j.lmot.2025.102131

*Wang, F., Zhou, X., Li, K., Cheung, A. C., & Tian, M. (2025b). The effects of artificial intelligence-based interactive scaffolding on secondary students’ speaking performance, goal setting, self-evaluation, and motivation in informal digital learning of English. *Interactive Learning Environments*, 1-20. https://doi.org/10.1080/10494820.2025.2470319

*Wang, G. L., Shakibaei, G., & Çakmak, F. (2025c). Examining the impact of gamified mobile and AI‐assisted language learning on academic integrity, creative trait motivation and writing performance. *British Educational Research Journal*. https://doi.org/10.1002/berj.4190

*Wang, Z., Zou, D., Zhang, R., Lee, L. K., Xie, H., & Wang, F. L. (2025d). ChatGPT-enhanced self-regulated learning in programming education: impacts on motivation, self-efficacy, and learning outcomes. *Interactive Learning Environments*, 1-26. https://doi.org/10.1080/10494820.2025.2559919

*Wei, L. (2023). Artificial intelligence in language instruction: impact on English learning achievement, L2 motivation, and self-regulated learning. *Frontiers in psychology, 14*, 1261955. https://doi.org/10.3389/fpsyg.2023.1261955

*Wu, T. T., Lee, H. Y., Li, P. H., Huang, C. N., & Huang, Y. M. (2024). Promoting self-regulation progress and knowledge construction in blended learning via ChatGPT-based learning aid. *Journal of Educational Computing Research, 61*(8), 1539-1567. https://doi.org/10.1177/07356331231191125

*Yıldız, T. A. (2023). The impact of ChatGPT on language learners’ motivation. *Journal of Teacher Education and Lifelong Learning, 5*(2), 582-597. https://doi.org/10.51535/tell.1314355

*Yilmaz, R., & Yilmaz, F. G. K. (2023). The effect of generative artificial intelligence (AI)-based tool use on students' computational thinking skills, programming self-efficacy and motivation. *Computers and Education: Artificial Intelligence, 4*, 100147. https://doi.org/10.1016/j.caeai.2023.100147

*Yuan, L., & Liu, X. (2025). The effect of artificial intelligence tools on EFL learners' engagement, enjoyment, and motivation. *Computers in Human Behavior, 162*, 108474. https://doi.org/10.1016/j.chb.2024.108474

*Zhang, L., Li, L., Jiang, J., & Zou, B. (2025a). Exploring the Impact of Diverse Feedback Sources on Learners’ Performance, Motivation, and Preference in a Translation Course: Tutor, Peer, and GPT Insight. *Thinking Skills and Creativity*, 102042. https://doi.org/10.1016/j.tsc.2025.102042

*Zhang, Q., Siraj, S. B., & Abdul Razak, R. B. (2025b). Effects of AI chatbots on EFL students’ critical thinking skills and intrinsic motivation in argumentative writing. *Innovation in Language Learning and Teaching*, 1-29. https://doi.org/10.1080/17501229.2025.2515111

*Zhang, Y. (2024, September). Artificial Intelligence in language instruction: Impact on English learning achievement and L2 motivational self-system. In *Proceedings of the International CALL Research Conference* (Vol. 2024, pp. 321-326). https://doi.org/10.29140/9780648184485-48

*Zhou, C., Ren, T., & Lang, L. (2025). The impact of AI-Based adaptive learning technologies on motivation and engagement of higher education students. *Education and Information Technologies*, 1-18. https://doi.org/10.1007/s10639-025-13646-x
